# Deep learning-based bimodal speech and facial expression recognition of miners’ unsafe emotions

**DOI:** 10.1371/journal.pone.0348906

**Published:** 2026-05-15

**Authors:** Ying Lu, Zihao Zhao, Liang Yan, Xinyv Shi

**Affiliations:** 1 School of Resource and Environmental Engineering, Wuhan University of Science and Technology, Wuhan, Hubei, China; 2 Hubei Industrial Safety Engineering Technology Research Center, Wuhan, Hubei, China; 3 School of Management, Fudan University, Shanghai, China; 4 Hubei Chemical Safety Association, Wuhan, Hubei, China; Firat Universitesi, TÜRKIYE

## Abstract

Under the influence of unsafe emotions, miners’ ability to perceive risks is hindered, which can easily lead to decision-making errors and safety accidents. To recognize unsafe emotions exhibited by miners during operations, this study proposes a deep learning-based bimodal framework that integrates speech and facial expression features. A convolutional neural network (CNN) combined with a bidirectional long short-term memory (Bi-LSTM) network is employed to model local spectral patterns and temporal dependencies in speech signals, and ShuffleNet-V2 is used to capture deep facial features. In addition, three feature enhancement strategies are proposed to improve the generalization ability of the model. By constructing a dataset containing five categories of miners’ unsafe emotions for network training, the model achieves a mean recognition accuracy of 85.56%. Furthermore, we conducted a preliminary field test of the bimodal model in a real mining environment. The results provide preliminary evidence of its potential applicability in real-world mining conditions.

## 1 Introduction

Mineral resources are the cornerstone of stable energy supply and sustainable economic development [1–3]. Nevertheless, the persistent occurrence of safety accidents in the mining sector constitutes a critical global challenge. In China, statistical data has indicated a 23.7% increase in the mortality rate per million tons in coal mines during 2023, thereby emphasising the necessity for more proactive safety interventions [4]. Recent interdisciplinary studies have demonstrated that miners’ unsafe behaviour is significantly driven by their emotional states [5–6]. Specifically, negative emotions act as a root cause for psychological instability, leading to impaired risk perception and decision-making errors [7–8]. In the high-pressure, harsh environments of underground mines, characterised by noise, dust, and isolation, miners are highly susceptible to anxiety, irritability, and burnout [9–12]. As indicated by previous research, miners experiencing negative emotional states have been shown to be 17 times more likely to incur injuries than those in a positive emotional state [6]. Consequently, the real-time recognition of miners’ unsafe emotions is imperative for the prediction of potential safety breaches and the prevention of accidents.

Against this background, the need for effective, objective, and scalable perception technologies to monitor miners’ emotional states has become increasingly evident. Recent studies have demonstrated the strong representational capacity of deep learning models across diverse industrial perception tasks, including medical image segmentation [13], vision transformer-based visual understanding [14], and CNN-driven speech recognition under low-resource conditions [15]. By contrast, the traditional safety inspection method for miners’ emotion still relies heavily on manual observation, which is inherently inefficient and subjective [16].

Therefore, an increasing number of deep learning-based emotion recognition models have been applied in safety-critical fields and industrial settings, including mining operations. Early studies predominantly focused on facial expression recognition, leading to the development of miner-specific models such as miniXception [17]. However, these unimodal approaches are highly sensitive to common disturbances in underground tunnels, including dust interference, insufficient lighting, and physical occlusions [18–19]. Alternative methods employing physiological sensors, such as electroencephalogram (EEG) [20–22] or electrodermal activity (EDA) [23–24], have the capacity to provide objective data. However, these methods are costly, uncomfortable for prolonged use, and difficult to deploy at scale in harsh mining operations [25].

To overcome the limitations of unimodal emotion recognition, recent studies have increasingly turned to multimodal frameworks that integrate complementary information sources [26–27]. However, extant multimodal frameworks generally employ power-intensive Transformer architectures [28] or rely on increasingly complex model designs to enhance prediction performance [29]. While such architectures demonstrate superior representation capability, they typically incur substantial computational and memory overhead, leading to high latency and energy consumption. Several recent studies have reported that Transformer-based multimodal emotion recognition models require significantly larger parameter sizes and longer inference times compared with lightweight CNN-based alternatives, which limits their deployment on edge devices and real-time monitoring systems [30–31]. In the mining environment, such computational demands pose a significant practical obstacle due to the severe constraints on computing resources [25]. Consequently, there is a pressing need for lightweight multimodal architectures that can achieve a favorable balance between recognition accuracy and computational efficiency, ensuring stable operation under harsh mining conditions.

Furthermore, while existing studies provide valuable insights into multimodal emotion recognition performance under controlled conditions, most publicly available datasets are collected in laboratory or semi-controlled environments, which fail to reflect the complex and dynamic characteristics of real-world mining scenarios [25,27]. Variations in background noise, illumination changes, visual occlusions, and individual behavioral differences can substantially degrade model performance when transferred to real-world settings [19,32–34]. Therefore, field experiments conducted in actual mining environments are essential for objectively assessing model robustness and practical applicability. Moreover, systematically analyzing the influence of environmental factors, such as sound intensity, distance, and visual occlusion, on recognition accuracy can provide critical evidence for targeted model optimization and deployment strategies.

A review of the current literature reveals three main research gaps. First, existing miner emotion recognition models primarily rely on facial expressions, making them vulnerable to environmental disturbances. Second, many multimodal frameworks suffer from large parameter sizes and high computational costs, limiting their portability. Third, current approaches lack the application exploration under real-world mining conditions, and existing datasets fail to reflect emotional variability.

In order to explicitly address the above research gaps, this paper proposes a bimodal deep learning model that has been specifically optimised for mine safety. A hybrid Convolutional Neural Network (CNN) and Bidirectional Long Short-Term Memory (Bi-LSTM) is employed to extract time-domain and frequency-domain features from speech, while an enhanced ShuffleNet-V2 is utilised for facial expression analysis. In contrast to conventional large-scale networks, ShuffleNet-V2 was selected for its well-structured architecture, which was developed specifically for mobile and edge devices, thereby achieving a superior balance between accuracy and scalability.

The main contributions of this study are summarized as follows:

(1) A deep learning-based bimodal framework is proposed to recognize miners’ unsafe emotions by jointly modeling speech and facial expression information.
(2) Three feature enhancement strategies, including frequency masking, coordinate attention, and squeeze-excitation mechanisms, are introduced to improve model generalization under harsh mining conditions.
(3) A dedicated dataset of miners’ unsafe emotions is constructed, covering five emotion categories relevant to mining safety. The proposed model is validated through numerical experiments, indoor simulations, and real mining field tests, which provides preliminary evidence of its potential applicability in mine operation scenarios.

The remainder of this study is organized as follows: Sect 2 describes the methodology, including dataset construction, model architecture, and experimental procedure. Sect 3 presents the results from numerical, indoor, and field experiments. Sect 4 discusses the model’s performance and environmental influences. Finally, Sect 5 concludes the paper and outlines future research directions.

## 2 Methodology

**Fig 1:**
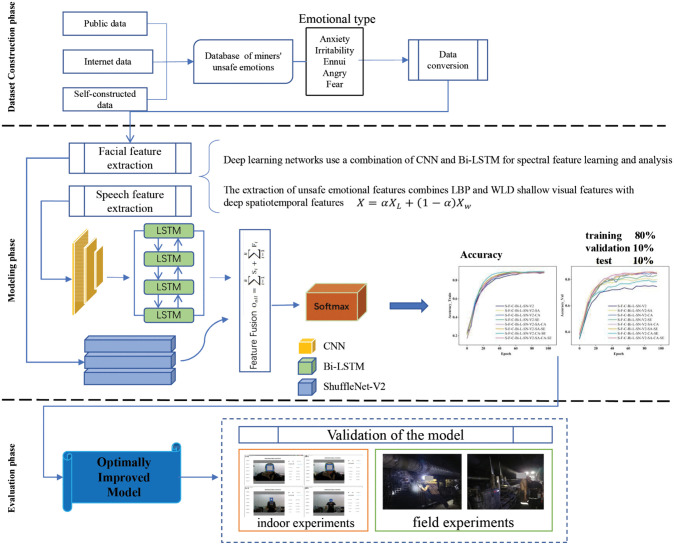
The main flow of the unsafe emotion recognition model. The individuals shown in this figure have given their written permission to publish their photographs under the CC BY 4.0 license.

The framework of the bimodal unsafe emotion recognition model proposed in this paper is shown in Fig 1, which firstly constructs speech and facial expression datasets separately, and then performs feature extraction on the speech and facial expression data respectively under the premise of guaranteeing the feature integrity, and finally outputs the emotion classification after feature fusion. We propose three types of feature enhancement methods to optimize the model. Specifically, for speech modality, MFCC features augmented with frequency masking are extracted and processed using a CNN–BiLSTM network with attention. For facial modality, LBP and WLD features are fused and analyzed using an improved ShuffleNet-V2 network with a squeeze-excitation mechanism. Through numerical experiments, indoor experiments and field experiments, the effectiveness of the model in different scenarios was preliminarily explored, and finally a bimodal miners’ unsafe emotion recognition system was constructed.

### 2.1 Ethics statement

This study was conducted in accordance with Item 32 of the Ethical Review Measures for Human Life Science and Medical Research issued by the National Health Commission of the People’s Republic of China (effective 27 February 2023) and qualifies for exemption from formal ethical review. The research involves only the collection of voice and facial expression data, poses no physical or psychological risk, and does not interfere with normal work activities.

All participants were fully informed of the study objectives, procedures, and data usage and provided written informed consent. Participation was voluntary, and participants could withdraw at any stage. The design and conduct of this study strictly adhere to the requirements stipulated for exempt research within the aforementioned regulation, particularly concerning respect for participant rights (as applicable to the data source), privacy protection, and data security.

### 2.2 Dataset

Most publicly available datasets are built on base emotions such as anger, surprise, frustration, happiness, fear and sadness, while it is difficult to accurately represent the categories of unsafe emotions such as anxiety and irritability, which have been mentioned in the study of miners’ emotions. Therefore, the dataset in this paper was collected in a variety of ways. Among them, the two categories of miners’ emotions, anger and fear, were collected from the CASIA database of the Chinese Academy of Sciences with 703 speech signals and 824 expression images. For the three emotion categories of anxiety, irritability and ennui, due to the difficulty of obtaining them from public databases, this study also developed an emotion dataset. The collected data were independently annotated by two co-authors following a predefined annotation protocol, without access to each other’s labeling results. To evaluate the consistency of manual annotations, inter-rater reliability was measured using Cohen’s Kappa coefficient for both voice and image modalities. The Cohen’s Kappa coefficient for the voice modality was 0.712, and for the image modality was 0.719, indicating substantial agreement between the two annotators. The confusion matrices of the annotations for the voice and image modalities are presented in Tables 1 and 2, respectively.

**Table 1:** Confusion matrix of speech emotion categories based on manual annotation.

Voice	Anxiety	Irritability	Ennui	Total (Annotator 1)
Anxiety	312	48	42	402
Irritability	45	318	39	402
Ennui	36	33	397	466
Total (Annotator 2)	393	399	478	1270

**Table 2:** Confusion matrix of facial emotion categories based on manual annotation.

Image	Anxiety	Irritability	Ennui	Total (Annotator 1)
Anxiety	358	52	46	456
Irritability	48	331	42	421
Ennui	39	35	460	534
Total (Annotator 2)	445	418	548	1411

For the annotations with differences, a consensus was reached through group discussion and the issues were resolved. A database of miners’ unsafe emotions containing 1,736 voice signals and 1,981 facial expression images was finally obtained after the labeling of emotion categories, and the detailed information of unsafe emotions in each category is shown in Table 3. The dataset was randomly stratified and divided into training, validation and test sets with a ratio of 80%, 10% and 10%, respectively.

**Table 3:** Database details of miners’ unsafe emotions.

	Category	Number of voices	Number of images	Total
Emotional type	Anxiety	315	362	Voice signal: 1736 Expression image: 1981
	Irritability	318	334	
	Ennui	400	461	
	Anger	390	497	
	Fear	313	327	

All data were obtained through legal channels or generated through observation without interfering with normal work activities. Data collection and analysis strictly complied with the terms and conditions of the respective data sources and adhered to Item 32 of the Ethical Review Measures for Human Life Science and Medical Research issued by the National Health Commission of the People’s Republic of China (effective 27 February 2023). Personal privacy was fully protected throughout the study.

### 2.3 Modeling of bimodal recognition of miners’ unsafe emotions

#### 2.3.1 Speech emotion recognition.

Speech emotion recognition consists of two parts, first extracting audio features from audio data and then analyzing it using deep learning networks.

Audio feature extraction is the key step in speech emotion recognition. Since emotions can be expressed through the speaker’s pronunciation, pitch, intensity, and many other acoustic features, proper selection of audio features has a significant impact on the performance of speech emotion recognition. Mel Frequency Cepstrum Coefficient (MFCC) was proposed by Davis [35], and is a widely used feature in the field of speech recognition.The MFCC is a powerful audio feature that captures the speech signal’s unique features for more accurate emotion classification and is the core of various speech emotion recognition studies. The audio data is first pre-emphasized, then the signal is processed in frames, multiplying each frame by a Hamming window to increase the continuity of the left and right ends, the frequency domain signal of each frame is obtained after FFT, the logarithmic spectrum is computed, and a bank of 64 Mel filters ranging from 20 to 8,000 Hz is applied to the frequency domain signal, the logarithmic operation is performed on the outputs of the filters, the dynamic range is suppressed, and finally the output of the filter is Arrange the output of the previous step to form the entire Mel spectrogram of the speech signal.

Before inputting the network, this paper employs the frequency masking method from SpecAugment to augment the spectrogram. Since it operates directly on the spectrogram without requiring additional audio data, this method is highly straightforward and computationally inexpensive. Furthermore, during augmentation, it can randomly remove certain frequency channels from the spectrogram of speech signals to simulate noise or damage. This enables the miner speech recognition module to better learn speech signal features, thereby enhancing model performance.

CNN + Bi-LSTM model is a deep learning model commonly used for emotion recognition tasks. It combines CNN and Bi-LSTM to fully capture contextual information in the text and model local features. Bi-LSTM is able to handle longer text sequences and maintains the ability to model long-distance dependencies. Combining these two models can simultaneously consider the spatio-temporal features of the miner’s emotional state and improve the model’s ability to understand the miner’s emotional state.The CNN structure used in this paper is shown in Table 4. Although CNN and Bi-LSTM perform well in image and sequence data processing, it may not be possible to fully capture the features of all emotional states using only CNN + Bi-LSTM. Different emotional states may exhibit different patterns and differences. Therefore, the introduction of Attention module in the miner’s emotion classification model can be used to assign weights according to the different impacts of input features on the output results, and improve the feature extraction ability of the network model by differentiating the weights.

**Table 4:** Parameter settings of CNN.

	Layer	KSize	Stride	Repeat
Conv	Convolution	5 × 5	2	3
	MaxPool	2 × 2	2	
	BN			
	ReLU			
	Dropout			

The Attention mechanism is modeled after the efficient information processing capability of the human visual system, which can quickly locate and focus on the key areas of the observed objects, and assign higher weights to the important targets, so as to achieve efficient information screening and recognition in complex scenes. Currently, this mechanism has been widely used in machine translation, text summarization, speech recognition and other tasks, and has become an indispensable and efficient module in deep learning models, especially in dealing with long sequence dependencies, cross-modal alignment and complex scene understanding and other scenarios, showing significant advantages [36]. Hou et al. [37] proposed a novel efficient coordinate attention mechanism, which integrates both horizontal and vertical positional information from feature maps into channel attention. This innovation enables mobile networks to capture extensive spatial information without excessive computational overhead. The proposed attention mechanism effectively captures long-range dependencies in speech features, dynamically adjusting the importance of different positions within feature maps. By assigning higher weights to emotion-related information, it achieves more precise target localization and enhances the contribution of emotional components to final recognition outcomes.

#### 2.3.2 Facial emotion recognition.

Facial emotion recognition comprises two main components: feature extraction and network analysis. For feature extraction, we employ a fusion of Local Binary Patterns (LBP) and Weber Local Descriptor (WLD) algorithms. LBP captures the edge direction information of images, while WLD focuses on the local intensity information. After extracting LBP and WLD features separately, we obtain the combined features by setting weighted fusion coefficients, as described in (1). These features are then input into the ShuffleNet-V2 network for analysis.


$$\begin{array}{c} X=\alpha X_{L}+(\text{1}-\alpha )X_{w} \end{array}$$ (1)

where $X_{L}$ is the LBP feature, $X_{w}$ is the WLD feature, and X is the hybrid feature

ShuffleNet V2 is an efficient neural network designed for high computational performance, accuracy, and lightweight deployment. The ShuffleNet-V2 network reduces the computational complexity of the model by stacking the compact operator deep convolution, and the convolution blocks stage2, stage3, and stage4 inside the network are composed of two parts: the first part is the first DownBlock in each stage. Since this module needs to downsample and enhance the dimension, the input information is directly copied into two copies, and the feature extraction is performed through the two branches of Branch1 and Branch2, respectively, after which the extracted feature information is spliced along the channel dimension, and finally the channels of the stacked feature maps are reordered through the Channel Shuffle to realize the feature fusion between the two branches. of the feature fusion. The second part is a number of BasicBlock in each stage, firstly, the number of channels of the input feature map is equally divided into two branches through Channel Spilt, the first path carries out residual linking to retain the original state, and the other branch realizes the extraction of feature information. Finally, the two branches are subjected to Channel Dimension Split as well as Channel Shuffle operation, so that the shallow feature information is effectively transferred to the deep network to achieve the effect of feature reuse.

To optimize the ShuffleNet-V2 network, we insert a squeeze-excitation (SE) mechanism at the end of the ShuffleNet unit. This enhancement addresses the issue in the bimodal emotion recognition model, where the facial emotion extraction module may not fully utilize the local feature information of images. The SE mechanism strengthens the directional features of facial expressions across five categories of unsafe emotions, thereby mitigating the problem of model overfitting.The overall network structure of the final ShuffleNet-V2 is shown in Table 5.

**Table 5:** Network structure of ShuffleNet-V2.

Layer	KSize	Stride	Repeat
Conv1	3 × 3	2	1
MaxPool	3 × 3	2	1
Stage2		2	1
		1	3
Stage3		2	1
		1	7
Stage4		2	1
		1	3
SENet			1
Conv5	1 × 1	1	1
GlobalPool	7 × 7		

#### 2.3.3 Feature fusion.

The speech and facial expression features of miners’ unsafe emotions are inherently sequential and often exhibit correlations. Therefore, during model construction, feature-level fusion of speech and facial expression features is employed to achieve multimodal information complementarity, leading to more accurate emotion assessment. The concatenation operation effectively preserves the distinct unsafe emotion information across different modalities [38]. Assuming that the speech feature vector group $O_{speech}=\left[S_{\text{1}},S_{\text{2}},\ldots S_{k}\right]$ and the expression feature vector group $O_{face}=\left[F_{\text{1}},F_{\text{2}},\ldots F_{k}\right]$ are obtained after feature extraction, then the fused feature vector group $O_{all}=\left[A_{\text{1}},A_{\text{2}},\ldots A_{k}\right]$ is obtained after concatenation, and finally the classification result is obtained by inputting into the softmax layer as shown in (2).


$$\begin{array}{c} O_{all}=[O_{speech};O_{face}] \end{array}$$ (2)

where [;] denotes the concatenation operation.

### 2.4 Experimental program

We designed a series of numerical experiments to validate the performance of our proposed speech feature extraction method, facial expression feature extraction method, and the bimodal miner’s unsafe emotion recognition model based on the feature layer fusion strategy, as well as to test the generalization ability of the model through two field experiments.

#### 2.4.1 Numerical experiments.

The relevant configuration of the experimental computer is Intel Core i5-9300H CPU, 16GB RAM, NVIDIA GeForce GTX 1660Ti, the experimental environment is Windows, the programming language is Python, and the deep learning framework is Pytorch. Our models were trained using a mini-batch size of 16 and categorical cross-entropy as a loss function. The Adam optimization algorithm for the initial learning rate of 0.001 and 100 epochs. To avoid the effect of overfitting, we employed an early stopping mechanism, which terminates training if the validation loss fails to improve for 10 consecutive epochs.

In order to verify the effectiveness of the enhancement strategies for speech and expression unsafe emotion features in the bimodal miner’s unsafe emotion recognition model, seven different improvement strategies are formulated for numerical experiments by combining three different types of network modules using S-F-C-Bi-L-SN-V2 as the baseline model, as shown in Table 6. To improve the robustness and generalizability of the experimental results, repeated random sub-sampling validation was conducted. Specifically, the dataset was randomly stratified and divided into training, validation, and test sets with a ratio of 80%, 10%, and 10% for five independent runs. Each model (baseline model and seven improved models) was implemented and evaluated under the same conditions, including identical hardware, software, hyperparameters, and the same five sets of stratified data partitions, and the final results were reported as the mean±standard deviation across all runs.

**Table 6:** Details of the numerical experimental program for different improvement strategies.

	Improvement program	Database	Evaluation index
Baseline model	S-F-C-Bi-L-SN-V2	Database of miners’ unsafe emotions	Accuracy, F1-Score, Loss Value
Improved models	S-F-C-Bi-L-SN-V2 + CA		
	S-F-C-Bi-L-SN-V2 + SA		
	S-F-C-Bi-L-SN-V2 + SE		
	S-F-C-Bi-L-SN-V2 + SA + CA		
	S-F-C-Bi-L-SN-V2 + SA + SE		
	S-F-C-Bi-L-SN-V2 + CA + SE		
	S-F-C-Bi-L-SN-V2 + SA + CA + SE		

#### 2.4.2 Indoor and field validation experiments.

In order to further verify the effectiveness of the improved optimal model for miners’ common unsafe emotion recognition, indoor experiments and field experiments are set up to analyze the performance of the model in different scenarios. Meanwhile, to facilitate the experiments, a prototype bimodal miner’s unsafe emotion recognition system for preliminary practical tests was developed using Python. The system can use audio and camera to realize emotion recognition. Due to the limitation of conditions, the indoor experiment chooses to use audio and camera monitoring for unsafe emotion recognition test, and the field experiment chooses to use audio and images for unsafe emotion recognition test. All subjects have signed the informed consent form for the experiment.

(1) **Indoor simulation program**

According to the improvement strategy in the previous section, the indoor experimental program was designed as shown in Table 7. Aiming at the biggest possible influencing factors of the emotion recognition model in the actual process: sound intensity and facial expression capture degree, different simulation scenarios are formed by the subject controlling the speaking voice size and the distance of the speaker. The experimental environment is an indoor laboratory of Wuhan University of Science and Technology, and the experiment is carried out in an environment with no construction noise, people walking around and other disturbances, and the sound intensity is controlled by the YSD130 noise detector. To ensure experimental fairness, all tests used the proposed model (S-F-C-Bi-L-SN-V2 + SA + CA + SE) with identical network backbone, pre-trained weights, data preprocessing, hyperparameters, and test environment. The sole variable was the input modality, with the unimodal speech test enabling only the speech branch with masked facial input, the unimodal facial test enabling only the facial branch with masked speech input, and the bimodal test activating both branches simultaneously.

**Table 7:** Indoor experimental program under different sound intensity and distance conditions.

	Model	Sound intensity (dB) + Distance (m)
1	S-C-Bi-L + SA + CA	50 + 0.5
2		50 + 1.0
3		60 + 0.5
4		60 + 1.0
5	F-SN-V2 + SE	50 + 0.5
6		50 + 1.0
7		60 + 0.5
8		60 + 1.0
9	S-F-C-Bi-L-SN-V2 + SA + SE + CA	50 + 0.5
10		50 + 1.0
11		60 + 0.5
12		60 + 1.0

(2) **Field experiment program**

We conducted field experiments on the optimal bimodal miners’ unsafe emotion recognition model selected from numerical and indoor experiments to verify the model’s real-world emotion recognition effect. Three experimental subjects covering different attribute information are selected, and the participant information is shown in Table 8. The voice and expression data were captured and recorded by camera, and then processed by audio editing software to obtain the measured data set for emotion recognition, and finally analyzed using the established bimodal miner unsafe emotion recognition model.

**Table 8:** Personal information of experimental participants.

	Height (cm)	Weight (kg)	Age	Marital status	Level of education	Job type	Seniority
A	170	65	25	Unmarried	Junior college	Mechanical electrician	3
B	173	70	36	Married	Junior high school	Mining workers	8
C	175	75	45	Married	Junior high school	Transporter	12

## 3 Results

### 3.1 Results of numerical experiments

**Fig 2:**
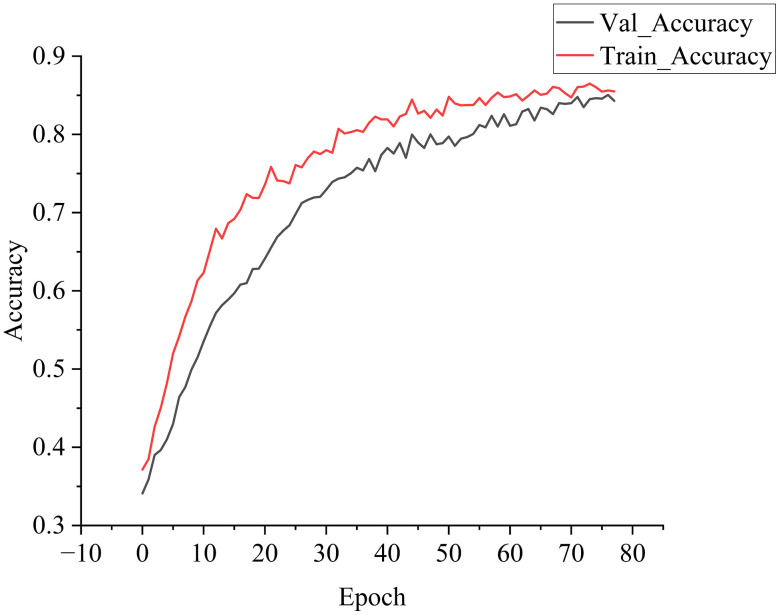
Comparison of Accuracy on Training and Validation.

**Fig 3:**
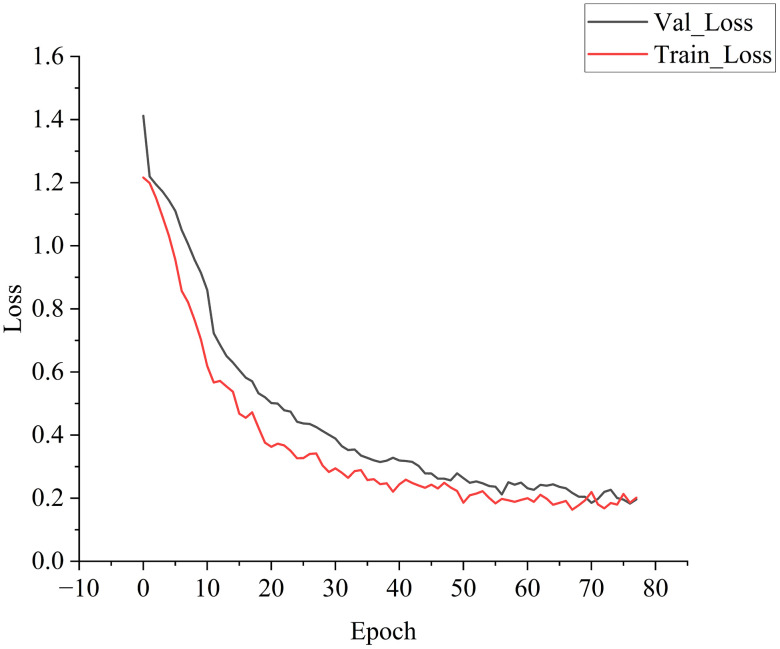
Comparison of Loss Rates on Training and Validation Sets.

Figs 2 and 3 illustrate the accuracy and loss curves for the training and validation sets from a representative run. As the number of epochs increased, the accuracy of both sets improved consistently while the loss values decreased. The early stopping mechanism was triggered at the 78th epoch after the validation loss remained stable for 10 consecutive iterations, indicating optimal model fitting without unnecessary computation.

Table 9 presents the ablation results of different modules. Specifically, we employed the baseline network (S-F-C-Bi-L-SN-V2) in the experiments, adding frequency masking, coordinate attention and squeeze-excitation mechanisms to the baseline architecture sequentially. It can be observed that the proposed model (S-F-C-Bi-L-SN-V2 + SA + CA + SE) achieves the best performance, with a mean accuracy of 85.56 ± 0.28% and a 95% confidence interval [85.21%, 85.91%]. Compared with the baseline (79.36 ± 0.20%), the full model improves accuracy by 6.20 percentage points. All improved models also outperform the baseline, with narrow confidence intervals indicating stable performance across different data splits.

**Table 9:** Ablation experimental results of different modules.

No	Model	Acc(%)	F1(%)	Loss	95% CI(%)
1	S-F-C-Bi-L-SN-V2	79.36 ± 0.20	78.19 ± 0.19	0.377 ± 0.002	[79.11%, 79.61%]
2	S-F-C-Bi-L-SN-V2 + CA	82.95 ± 0.15	82.71 ± 0.16	0.248 ± 0.002	[82.76%, 83.14%]
3	S-F-C-Bi-L-SN-V2 + SA	82.18 ± 0.17	81.56 ± 0.17	0.270 ± 0.002	[81.97%, 82.39%]
4	S-F-C-Bi-L-SN-V2 + SE	80.89 ± 0.17	81.15 ± 0.18	0.305 ± 0.002	[80.68%, 81.10%]
5	S-F-C-Bi-L-SN-V2 + SA + CA	85.02 ± 0.16	84.88 ± 0.16	0.238 ± 0.002	[84.82%, 85.22%]
6	S-F-C-Bi-L-SN-V2 + SA + SE	84.41 ± 0.17	84.12 ± 0.17	0.256 ± 0.002	[84.20%, 84.62%]
7	S-F-C-Bi-L-SN-V2 + CA + SE	83.10 ± 0.17	82.79 ± 0.17	0.270 ± 0.002	[82.89%, 83.31%]
8	S-F-C-Bi-L-SN-V2 + SA + CA + SE	85.56 ± 0.28	85.20 ± 0.27	0.223 ± 0.003	[85.21%, 85.91%]

To assess the statistical significance of these improvements, we performed paired t-tests based on the five independent runs using accuracy as the evaluation metric. As summarized in Table 10, every improved model shows a statistically significant gain over the baseline (all p < 0.001). Furthermore, the proposed model also achieves a significant improvement over the second-best model (S-F-C-Bi-L-SN-V2 + SA + CA), with a mean difference of 0.54 percentage points (t = 2.93, p = 0.043). These results confirm that each proposed enhancement strategy contributes meaningfully to recognition accuracy, and that the integrated model provides the best overall performance with statistical reliability.

**Table 10:** Statistical significance analysis based on Accuracy.

Comparison	Mean Difference (%)	t-value	p-value
+CA vs Baseline	3.59	22.2	<0.001
+SA vs Baseline	2.82	16.5	<0.001
+SE vs Baseline	1.53	11.8	<0.001
+SA + CA vs Baseline	5.66	34.1	<0.001
+SA + SE vs Baseline	5.05	29.7	<0.001
+CA + SE vs Baseline	3.74	22.9	<0.001
+SA + CA + SE vs Baseline	6.20	30.95	<0.001
+SA + CA + SE vs Second-best(+SA + CA)	0.54	2.93	0.043

### 3.2 Results of indoor experiments

**Fig 4:**
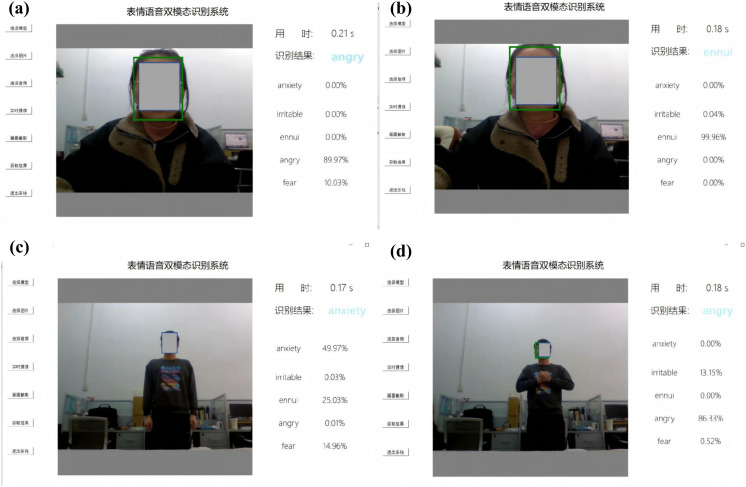
Emotion Detection Recognition Results: (a) 50 dB + 0.5 m; (b) 60 dB + 0.5 m; (c) 50 dB + 1.0 m; (d) 60 dB + 1.0 m (Facial regions are blurred for privacy protection; the original unblurred images were used for model training and evaluation).

We conducted indoor experiments according to the program in Table 7. Fig 4 presents the system interface during real-time recognition. The model demonstrated high efficiency, with an average inference latency of approximately 0.20s per frame under various environmental configurations. It should be noted that all facial images presented in this manuscript have been blurred to protect the privacy of the participants. However, the original unblurred images were used during the training and evaluation stages of the experiments. Therefore, the blurring of images in the manuscript does not affect the reproducibility or validity of the experimental results.

The results in Table 11 show that the improved bimodal miner’s unsafe emotion recognition model has the best experimental performance, with the highest recognition accuracy at a sound intensity of 60 dB and a distance of 0.5 m. Compared to unimodal speech and facial expression models in the same scenario, the bimodal approach improved recognition accuracy by 6.38% and 7.87%, respectively. For the simulation scene (sound intensity of 50dB, distance of 1.0m), which has the lowest accuracy among the measured scenes of the bimodal model, outperforming the unimodal speech and expression models by 4.31% and 4.68%, respectively.

**Table 11:** Results for different sound intensity and distance conditions.

	Model	Sound intensity (dB) + distance (m)	Accuracy
1	S-C-Bi-L + SA + CA	50 + 0.5	82.15
2		50 + 1.0	81.78
3		60 + 0.5	85.69
4		60 + 1.0	83.42
5	F-SN-V2 + SE	50 + 0.5	82.95
6		50 + 1.0	81.41
7		60 + 0.5	84.20
8		60 + 1.0	80.05
9	S-F-C-Bi-L-SN-V2 + SA + SE + CA	50 + 0.5	88.45
10		50 + 1.0	86.09
11		60 + 0.5	92.07
12		60 + 1.0	89.13

### 3.3 Results of field experiments

**Fig 5:**
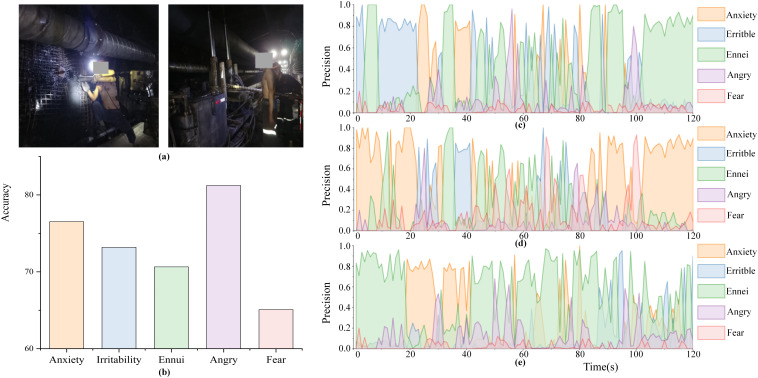
Field experiment results: (a) miner’s work site; (b) average recognition accuracy; (c) temporal distribution of unsafe emotions for miner A; (d) miner B; (e) miner C. The individuals shown in this figure have given their written permission to publish their photographs under the CC BY 4.0 license.

In order to preliminarily explore the practical feasibility of the model in real-world mining environments, we carried out field experiments with the informed consent of all participants. As shown in Fig 5(a), we collected audiovisual data from three miners during their daily work. After processing, we obtained speech signals and facial expression fragments with obvious emotional characteristics, and used the optimal model to recognize the emotional state of the three miners.

The average recognition accuracy and distribution of five types of unsafe emotions among the three miners are shown in Fig 5(b) and Fig 5(c)–Fig 5(e). The highest recognition accuracy for the anger emotion category was 81.25%, followed by anxiety and irritability with 76.56% and 73.23%, respectively. Ennui and fear had the lowest recognition accuracies of 70.67% and 65.09%, respectively.

## 4 Discussion

### 4.1 Optimally improved model

The experimental results demonstrate that the overall recognition performance of the improved network is consistently superior to that of the baseline model across multiple random data splits. In particular, the proposed model (S-F-C-Bi-L-SN-V2 + SA + CA + SE) achieves the best performance, with an average accuracy of 85.56 ± 0.28% and an F1-score of 85.20 ± 0.27%. Moreover, the relatively small standard deviation and narrow confidence interval demonstrate that the proposed model maintains stable performance under different data partitioning conditions. A comparative analysis of the eight models shows that all three feature enhancement strategies contribute to performance improvement to varying degrees. Among them, the coordinate attention mechanism provides the most significant contribution, followed by frequency masking and squeeze-excitation mechanisms. On average, the coordinate attention module improves accuracy by approximately 3.6% compared with the baseline, while frequency masking and squeeze-excitation bring improvements of approximately 2.8% and 1.5%, respectively.

To validate the effectiveness of the proposed method, we compared it with several state-of-the-art approaches. The accuracy results we compared were all reported in these research works. Table 12 demonstrates that on the Fer2013 dataset, the bimodal model achieved an average accuracy of 84.51%, representing a 0.21% improvement in recognition accuracy over the unimodal MiniXception network. Although there remains a certain performance gap compared to Transformer-based architectures, the proposed model achieves a favorable balance between recognition accuracy and computational efficiency, and has the potential to be applied in underground mining edge monitoring scenarios. Transformer-based models typically require higher computational resources, which limits their deployment in underground mining environments with constrained computing power. On the Ravdess dataset, the bimodal model attained an average accuracy of 88.17%, surpassing the state-of-the-art method by 0.27%. It also demonstrated significant performance advantages over other lightweight models.

**Table 12:** Performance comparison of different methods.

Source	Method	Dataset	Acc
[17]	MiniXception	Fer2013	84.3
[31]	Transformer with graph attention	Fer2013	90.4
[39]	CNN and BiLSTM are used by Transfer Learning	RAVDESS	80.08
[40]	BiLSTM-multi-head attention	RAVDESS	82.42
[41]	Lightweight CNN with Uncertainty Learning	RAVDESS	81.9
[42]	3DCNN	RAVDESS	87.9
[43]	VCAN	RAVDESS	75.9
[44]	Lightweight VGGNet	RAVDESS	86.25
Ours	S-F-C-Bi-L-SN-V2 + SE + SA + CA	Fer2013 RAVDESS	84.51 88.17

### 4.2 Application analysis of the improved model

(1) **The effect of sound intensity and distance in practical application of the model**

**Fig 6:**
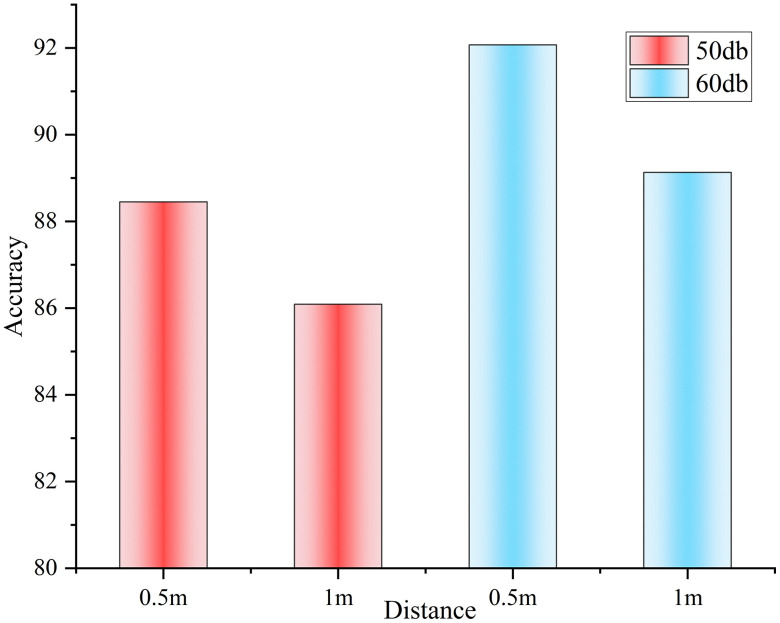
Effect of sound intensity and distance on accuracy.

The results in Table 11 show that at a sound intensity of 50 dB, the accuracy decreases by 2.36% for every 0.5 m increase in distance, and at a sound intensity of 60 dB, the accuracy decreases by 2.94% for every 0.5 m increase in distance. Similarly, at a distance of 0.5m, for every 10dB increase in sound intensity, the accuracy rate increases by 3.62%. At a distance of 1.0m, for every 10dB increase in sound intensity, the accuracy rate increases by 3.04%. From the above series of data changes combined with Fig 6, it can be found that the change of sound intensity contributes more to the accuracy of the improved bimodal model for emotion recognition than the change of distance, i.e., the accuracy is more significantly affected by the size of sound intensity. This phenomenon has received limited attention in previous studies, and our next research work can further evaluate how the interaction of audio and visual information affects emotion recognition accuracy under different sound intensity and distance conditions.

(2) **Other areas where improvements can be made in practical application**

From the results in Fig 5(b), the optimal bimodal model achieves the recognition of different categories of unsafe emotions. The low recognition accuracy of ennui and fear, 70.67% and 65.09%, respectively, may overlap with the features of anger emotions, making it difficult to differentiate, and may also be related to external environmental factors such as the noise generated by the operation of the facilities at the work site of the miners and the wearing of helmets, which reduces the accuracy of the model. Some studies [32–34] have pointed out that the underground environment of coal mines is characterized by dim lighting, dust interference, obstruction, position changes, multiple targets, and other complex factors, which can significantly reduce recognition accuracy. These findings also provide a basis for future improvements of the model.

In addition, although the field data cover typical mining interference factors, due to the relatively small number of participants, the experimental results can be regarded as a preliminary verification of the application potential of the model. Nevertheless, this exploratory field experiment offers a preliminary attempt to evaluate the model under real-world mining conditions. Future research needs to carry out more large-scale field research in more mining areas to further verify the applicability and long-term stability of the model in real-world mining scenarios.

## 5 Conclusion and future research work

The present study proposes a deep learning-based model for identifying unsafe emotions in miners. In the feature extraction process, three feature enhancement strategies are proposed: First, frequency masking is applied as part of the SpecAugment strategy to enhance speech spectrogram representations. The weight of emotional information is increased through the attention mechanism. The ShuffleNet-V2 network is optimised in combination with the SE mechanism. Subsequently, group comparisons were conducted using numerical experiments. The results of the study show that these three feature enhancement strategies contribute to performance improvements. The proposed model (S-F-C-Bi-L-SN-V2 + SA + CA + SE) achieves the best overall performance, with an average accuracy of 85.56 ± 0.28% and an F1-score of 85.20 ± 0.27%. The narrow confidence interval and low variance indicate that the proposed method exhibits stable performance across different data partitioning conditions.

It is noteworthy that, during real-world testing, it was discovered that sound intensity has a more significant impact on recognition accuracy – a phenomenon that has received limited attention in previous studies. Consequently, these issues merit further optimisation and exploration in subsequent research. Additionally, Future work will focus on expanding the dataset to include large-scale real-world mining data, exploring a more refined emotional classification system, and further validating the applicability and long-term stability of the model through more extensive field experiments. In addition, transformer-based cross-modal fusion strategies will be investigated to further improve robustness under extreme noise and occlusion conditions.
